# Comparison between Unilateral and Bilateral Ovarian Drilling in Clomiphene Citrate Resistance Polycystic Ovary Syndrome Patients: A Randomized Clinical Trial of Efficacy

**DOI:** 10.22074/ijfs.2015.4202

**Published:** 2015-04-21

**Authors:** Ziba Zahiri Sorouri, Seyede Hajar Sharami, Zinab Tahersima, Fatemeh Salamat

**Affiliations:** 1 Reproductive Health Research Center, Department of Obstetrics and Gynecology, Alzahra Hospital, Guilan University of Medical Sciences, Rasht, Iran; 2 Research Vice Chancellorship, Guilan University of Medical Sciences, Rasht, Iran

**Keywords:** Bilateral, Unilateral, Ovarian Induction, Polycystic Ovary Syndrome

## Abstract

**Background:**

Laparoscopic ovarian drilling (LOD) is an alternative method to induce ovulation in polycystic ovary syndrome (PCOS) patients with clomiphene citrate (CC) resistant instead of gonadotropins. This study aimed to compare the efficacy of unilateral LOD (ULOD) versus bilateral LOD (BLOD) in CC resistance PCOS patients in terms of ovulation and pregnancy rates.

**Materials and Methods:**

In a prospective randomized clinical trial study, we included 100 PCOS patients with CC resistance attending to Al-Zahra Hospital in Rasht, Guilan Province, Iran, from June 2011 to July 2012. Patients were randomly divided into two ULOD and BLOD groups with equal numbers. The clinical and biochemical responses on ovulation and pregnancy rates were assessed over a 6-month follow-up period.

**Results:**

Differences in baseline characteristics of patients between two groups prior to laparoscopy were not significant (p>0.05). There were no significant differences between the two groups in terms of clinical and biochemical responses, spontaneous menstruation (66.1 vs. 71.1%), spontaneous ovulation rate (60 vs. 64.4%), and pregnancy rate (33.1 vs. 40%) (p>0.05). Following drilling, there was a significant decrease in mean serum concentrations of luteinizing hormone (LH) (p=0.001) and testosterone (p=0.001) in both the groups. Mean decrease in serum LH (p=0.322) and testosterone concentrations (p=0.079) were not statistically significant between two groups. Mean serum level of follicle stimulating hormone (FSH) did not change significantly in two groups after LOD (p>0.05).

**Conclusion:**

Based on results of this study, ULOD seems to be equally efficacious as BLOD in terms of ovulation and pregnancy rates (Registration Number: IRCT138903291306N2).

## Introduction

The most common cause of anovulatory infertility is polycystic ovary syndrome (PCOS) (1,4). Induction of ovulation with clomiphene citrate (CC) is the first line of treatment in these patients (1,5,8). CC resistant is defined as failure to ovulate after receiving a maximum dosage of 150 mg per day for five days beginning on the third day of menstrual cycle (1,9) Laparoscopic ovarian drilling (LOD) is an alternative method to induce ovulation in these patients instead of administration of gonadotropins (1,2,10,14). Despite minimal morbidity associated with this method, LOD has some benefits. The benefits consist of the elimination of cycles monitoring, decreasing the risk of ovarian hyperstimulation syndrome (OHSS), multifetal pregnancy associated with gonadotropins (2,14,21), as well as occurring spontaneous ovulation in some patients without further treatments (16). Two disadvantages of LOD are the probability of tuboovarian adhesion (TOA) (12,22,31) and risk of premature ovarian failure (POF) (24,25). Reducing the potential damage to ovarian surface epithelium (OSE) leads to a significantly decreased risk for TOA and POF (24,25). A few studies have compared unilateral LOD (ULOD) and bilateral LOD (BLOD) and concluded that ULOD is equally efficacious as BLOD in inducing ovulation and achieving pregnancy besides minimizing the risk of adhesion and POF (24,25,32,34). Therefore, changing the usual method of LOD for both ovaries to only one ovary may minimize those risks. This study was done prospectively to compare the efficacy of ULOD versus BLOD in CC resistant patients in terms of ovulation and pregnancy rates. 

## Materials and Methods

**Fig.1:**
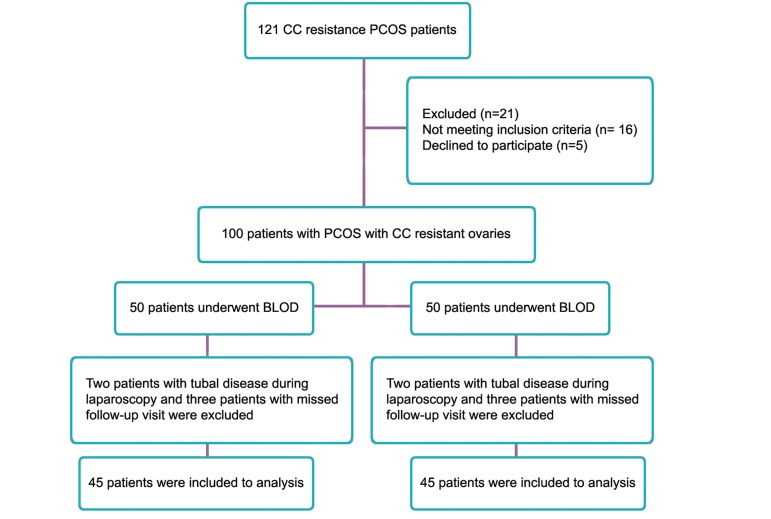
Flowchart of randomized clinical trial for comparing ULOD versus BLOD in CC resistance PCOS patients. ULOD; Unilateral laparoscopic ovarian drilling, BLOD; Bilateral laparoscopic ovarian drilling, CC; Clomiphene citrate and PCOS; Polycystic ovary syndrome.

This prospective parallel randomized clinical trial was conducted in Al-Zahra Hospital in Rasht, Guilan Province, Iran, from June 2011 to July 2012. Among PCOS women attending the infertility clinic with CC resistant ovaries, 121 patients with CC resistance PCOS were initially examined. Before laparoscopy, five patients had other endocrine abnormally, four patients had mechanical factors abnormally such as unilateral or bilateral tubal blockages in hysteroscopy (HSC), seven patients had concomitant male infertility, and five patients refused to participate in the study; therefore, 100 patients were included in this study (Fig.1). 

Given that few studies have been done in this field, this study was considered as a pilot study after considering the attrition coefficient, so an equal number (n=50) were allocated to each ULOD (group I) and BLOD (group II) groups. PCOS patients were diagnosed based on presence two out of three Rotterdam 2003 criteria, including: oligomenorrhea and/or anovulation, hyper androgenism (biochemical or clinical) and PCOS. We used transvaginal ultrasound to diagnose PCOS, after ruling out other causes, like congenital adrenal hyperplasia (CAH), Cushing syndrome, administration of androgen, and androgen secreting tumor (AST). CC resistant is defined as failure to ovulate after receiving a maximum dosage of 150 mg per day for five days beginning on the third day of menstrual cycle (1,9). All patients had normal hysterosalpingography and their partners had normal spermiogram using criteria of World Health Organization (WHO). Also all patients had normal uterus in ultrasound scan. Normal uterus was defined as normal size and shape with regular endometrium without any polyp or myoma. According to laparoscopic findings, patients with evidence of tubo-peritoneal diseases, such as tubal obstruction and peritoneal adhesion to tubes or ovaries and endometriosis were also excluded. Among 50 patients in group I undergoing ULOD, two patients were excluded because of tubal disease diagnosed during laparoscopy, and three patients were excluded due to the missed follow-up visit. Among 50 patients in group II, one patient was excluded because of endometriosis diagnosed during laparoscopy, and 4 patients were excluded due to the missed follow-up visit. Finally 45 patients in each group were included for analysis (Fig.1). The cycles of all patients were oligomenorrhea or amenorrhea. 

This study was approved by the Ethics Committee of Guilan University of Medical Sciences, Guilan, Iran. All patients provided a written informed consent before entering the study. A randomization list was generated using blocked sample randomization. The permuted block randomization method was used in order to give a block size of four. Assignment proceeds by randomly selecting one of the orderings and allocating the next block of subjects to groups according to the specific sequence. Prior to the laparoscopic procedure, all the patients were tagged in the changing room before entering the operation room by an operating room (OR) nurse, in a blocked randomization design, and the surgeon were not aware of the type of the tag, before entering the operation room. 

All 100 PCOS patients with CC resistance were randomly assigned into ULOD (group I) and BLOD (group II) groups. The group I, right ovary, and group II, both ovaries, underwent electrocauterization. We chose right ovary in ULOD because most of the studies have concluded that ovulation occurs more frequently (about 55% of the time) in the right ovary as compared with the left one, and oocytes from the right ovary have a higher potential for pregnancy (35). Besides the probability of adhesion is more in left ovary than right one (31). For all patients, triple puncture laparoscopy was done by a gynecologist. After establishing tubal patency with methylene blue, LOD was performed using unipolar diathermy needle (Karl Storz, Germany). The penetration was about in depth of 8 mm, a setting of 60W, and 5 points per ovary. Ovaries were cooled by normal saline immediately after cauterization, and about 300500 ml of normal saline was left in pelvic cavity for prevention of adhesion. In the cases of any complications during surgery such as anesthetic problems or injury to organs, the operation was discontinued and the cases were dropped out of the study. The variables, including: age, infertility duration, cycle characteristic (oligomenorrhea or amenorrhea), body mass index (BMI), follicle-stimulating hormone (FSH), luteinizing hormone (LH), and testosterone level on day 3 of spontaneous or induced menstruation, were assessed before and after laparoscopy. 

The day after laparoscopy, the women were asked to keep their menstrual calendar. If the patients started a menstrual period within 6 weeks after LOD, a blood sample for measurement of LH, FSH and testosterone levels was taken on days 2-3 of menstrual cycle. If spontaneous menstruation did not occur within 6 weeks, an intramuscular injection of 100 mg progesterone (Iran hormone, Iran) was prescribed. After excluding pregnancy, on days 2-3 of menstrual cycle, hormonal measurements were done. 

Ovulation was assessed on day 21 by measurement of progesterone in patients who had spontaneous menstruation. Progesterone level >3 ng/ mL is considered as ovulation. If there was no ovulation as evidenced by progesterone level or lack of menstruation, the patients was advised to use CC with starting dose of 50 mg/day up to 150 mg/day from days 3-7 that was monitored by ultrasound. Patients were followed-up until they conceived or 6 mount after LOD. The clinical, defined as menstrual resumption, spontaneous or induced, and biochemical, defined as FSH, LH and free testosterone levels before and after surgery, responses on ovulation and pregnancy rates were measured. In this study, pregnancy was defined as detection of fetal heart on transvaginal ultrasound. 

## Statistical analysis

Data were analyzed in IBM SPSS software, version 11.5. (SPSS, SPSS Inc., Chicago, IL, USA). We used descriptive and analytic statistics. For numeric variables, data were described as mean and standard deviation, while for categorical variables, data were shown as number and percentage. For statistical analysis, independent t test (two-tailed) was used to compare mean values between two groups, while paired t test was used to compare mean values of FSH, LH, and testosterone levels before and after LOD. Also Fisher’s exact test was used to compare relative proportions of variables between two groups. Differences were considered significant at p<0.05. 

## Results

Total of 100 patients with PCOS who underwent LOD were included in this study. These patients were divided into two groups of ULOD or BLOD equally. Among 50 patients in group I undergoing ULOD, two patients were excluded because of tubal disease diagnosed during laparoscopy, and three patients were excluded due to the missed follow-up visit. Among 50 patients in group II undergoing BLOD, one patient was excluded because of endometriosis diagnosed during laparoscopy, and 4 patients were excluded due to the missed follow-up visit. Finally 45 patients in each group were included for analysis (Fig.1). 

The baseline characteristics of the women in two groups are shown in table 1. There were no significant differences between patients of two groups in terms of clinical and endocrinologic characteristics and cycle history. After LOD, 30 (66.7%) of patient in group I and 32 (71.1%) in group II had spontaneous menstruation within 6 weeks. But this difference was not statistically significant (p=0.820). To induce menstrual period, an intramuscular injection of 100 mg progesterone was prescribed for remaining women (Table 2). 

The ovulation rate after the first menstruation (spontaneous or induced) was assessed with mid-lutealprogesterone level. Overall 27 (60%) patients in group I and 29 (64.4%) patients in group II had spontaneous ovulation. CC was used by starting dose of 50 mg/day up to 150 mg/day for 5 days from third day of cycle, while the findings showed that 11 (24.4%) women in group I and 11 (24.4%) women in group II ovulated successfully. 

There were no significant differences between two groups in term of spontaneous (p=0.82) or CC-induced (p=0.70) ovulation (Table 2). Fourteen women (31.1%) in group I and 18 women (40%) in group II were pregnant within 6 month of follow-up visit, but this difference was not statistically significant. In both groups, after LOD, means serum levels of LH (p=0.0001) and testosterone (p=0.001) were decreased significantly. 

Also there were no significant differences in means serum levels of LH (p=0.322), testosterone (p=0.079) and FSH (p=0.758) between two groups after drilling (Table 3). Two cases in group II were aborted after detection of fetal heart and one case of triplet was seen in group I. 

**Table 1:** Baseline characteristics of 90 CC-resistant PCOS patients prior to laparoscopy


Screening parameter	Group I (n=45)	Group II (n=45)	P value

Clinical			
**Mean age (Y)**	27.60±4.25	28.02±4.27	0.644
**Mean of infertility duration (Y)**	3.04±2.78	4.11±2.61	0.064
**Mean of menarche (Y)**	12.86±1.84	12.64±1.70	0.649
**BMI (%)**			
**>30**	35.6%	48.9%	0.286
**≤30**	64.4%	51.1%	
**Cycle history (%)**			
**Amenorrhea**	20%	13.3%	0.573
**Oligomenorrhea**	80%	86.7%	
**Endocrinologic: (mean)**			
**LH (IU/L )**	11.1±0.6	11.4±1.4	0.601
**FSH (IU/L )**	5.7±1.7	5.8±2.5	0.840
**Testosterone (pg/ml)**	1.7±0.8	1.9±1.3	0.455


CC; Clomiphene citrate, PCOS; Polycystic ovary syndrome, BMI; Body mass index, LH; Luteinizing hormone and FSH; Follicle stimulating hormone.

**Table 2:** Clinical response on ovulation and pregnancy rates in 90 CC-resistant PCOS patients after laparoscopy


	Group I n (%)	Group II n (%)	P value

Menstrual resumption			
Spontaneous	30(66.7)	32(71.1)	0.820
Induced	15(33.3)	13(28.9)	
**Ovulation rate**			
Spontaneous	27(60)	29(64.4)	0.828
Induced	11 (24.4)	11 (24.4)	0.715
**Pregnancy rate**	14(31.1)	18(40)	0. 350


CC; Clomiphene citrate and PCOS; Polycystic ovary syndrome.

**Table 3:** Comparison among mean serum levels of FSH, LH, and testosterone before and after LOD


Mean serum level		Before LOD	After LOD		P value

**FSH (IU/L)**	Unilateral	5.7±1.7	5.7	± 2.1	0.940
	Bilateral	5.8±2.5	6±2.6		0.577
**T test p value**		0.840	0.758		
**LH (IU/L)**	Unilateral	11.1±0.6	6.1	± 3.4	<0.001
	Bilateral	11.4±1.4	7±2.5		<0.001
**T test p value**		0.601	0.322		
**Testosterone (pg/ml)**	Unilateral	1.7±0.8	1.2	± 0.75	0.001
	Bilateral	1.9±1.3	1.5	± 1.7	0.001
**T test p value**		0.455	0.079		


LOD; Laparoscopic ovarian drilling, LH; Luteinizing hormone and FSH; Follicle stimulating hormone.

## Discussion

In this study, we have evaluated the effect of ULOD versus BLOD on the ovulation and pregnancy rates of 90 CC resistant PCOS patients. We found that there are no significant differences between groups in terms of ovulation and pregnancy rates.

PCOS women who are CC resistant can be treated with gonadotropins, but there are risks of OHSS and multiple pregnancies in this method. Also gonadotropines are expensive and time-consuming treatment requiring intensive monitoring. Surgical therapy is an alternative method for ovulation induction in these patients to overcome the disadvantages of gonadotropins (1, 2, 9, 14, 36, 37). Ovarian wedge resection surgery was an accepted method of ovulation induction over 40 years (38). However, it was abandoned because of adhesion formation (39-41). LOD was first described by Gjonnaess in 1984 (42).

The mechanism of LOD is similar to ovarian wedge resection surgery. Destruction of androgenproducing ovarian tissue leads to a decrease in the peripheral conversion of androgen to estrogen. Decreased serum levels of androgen and LH and increased FSH level have been demonstrated after ovarian drilling (40, 43, 44). A change in endocrine function converts the androgen-dominant intrafollicular environment to estrogenic one (45). It affects ovarian-pituitary feedback mechanism (46), so both local and systemic effects may induce ovulation in these patients. Due to ovulation and pregnancy success rates, mentioned in various studies, LOD is an accepted method for ovulation induction in CC resistant PCOS patients (25).

Two important potential adverse effects of LOD are peri-ovarian adhesions and reduced ovarian function (47, 48). The rate of peri-ovarian adhesion is very different in various studies, from 19 to 43%, and with greater damage to the ovaries, the risk become higher (42, 49-51). Furthermore POF is another concern of LOD that is dependent on the number of puncture made (>4-6) (52). Therefore, the risk of peri-ovarian adhesion and the rate of POF can be minimized by decreasing the number of punctures (24, 25).

The idea of ULOD instead of BLOD for minimizing these two side effects was first introduced by Ballen and Jacobs (53). They showed that ULOD can result in bilateral ovarian activity due to local cascade of growth factors, such as insulinlike growth factor-1 (IGF-1), which interacts with FSH, leading to a decrease in the serum LH concentration (53, 54).

Nowadays BLOD is a standard method of LOD. Few studies compared ULOD and BLOD and concluded that ULOD is as effective as BLOD and minimizes the risk of adhesion and POF (24, 25, 31-34, 54, 55).

In this study, after performing LOD, we found significant decreases in serum levels of LH and testosterone in both groups that were similar in both groups. Also there were no significant differences between groups in terms of ovulation and pregnancy rates. Youssef and Atallah (25) in 2007 evaluated 87 patients with ovulation failure as a result of PCOS who were randomly allocated into ULOD (n=43) and BLOD (n=44). In patients who ovulated after drilling, there was a significant fall in serum LH concentration, while ovulation, pregnancy and miscarriage rates were similar between both groups. Roy et al. (24) in 2009 evaluated the effect of ULOD versus BLOD in 22 patients. The clinical and biochemical responses on ovulation and pregnancy rates over a 1-year follow-up period were compared. They also evaluated tubo-ovarian adhesion rate during cesarean section or a second-look laparoscopy. They found no significant differences between two groups in terms of clinical and biochemical responses, ovulation and pregnancy rates, and tubo-ovarian adhesions. They concluded that ULOD may be a suitable option in CC resistant infertile patients of PCOS which can replace BLOD with the potential advantage of decreasing the chance of adhesion formation. Abdelhafeez et al. (55) in 2013 reported that ULOD is as effective as BLOD in terms of restoration of regular menstrual pattern and ovulation rate. Sunj et al. (31) in 2013 represented that the results of applied method can be improved when using less thermal energy in volumeadjusted ULOD in comparing to BLOD. 

## Conclusion

Based on the results of this study, ULOD seems to be equally efficacious as BLOD in terms of ovulation and pregnancy rates. 
